# Effectiveness and durability of solifenacin versus percutaneous tibial nerve stimulation versus their combination for the treatment of women with overactive bladder syndrome: a randomized controlled study with a follow-up of ten months

**DOI:** 10.1590/S1677-5538.IBJU.2016.0611

**Published:** 2018

**Authors:** Carlo Vecchioli-Scaldazza, Carolina Morosetti

**Affiliations:** 1 Division of Urogynecology, ASUR, Jesi, Italy; 2 Clinical Pathology, ASUR, Jesi, Italy

**Keywords:** Urinary Bladder, Overactive, Combined Modality Therapy, Solifenacin Succinate

## Abstract

**Purpose:**

To assess effectiveness and durability of Solifenacin (SS) versus tibial nerve stimulation (PTNS) versus combination therapy (PTNS + SS) in women with overactive bladder syndrome (OAB).

**Materials and Methods:**

105 women with OAB were divided randomly into three groups of 35 patients each. In group A women received SS, in group B women underwent PTNS, in group C women underwent combination of PTNS + SS. Improvements in OAB symptoms were assessed with OABSS questionnaire; patients’ quality of life was assessed with OAB-q SF questionnaire. Evaluation of effectiveness of treatments was performed with PGI-I questionnaire. OABSS and PGI-I were also assessed monthly for ten months.

**Results:**

All treatments were effective on symptoms. PTNS showed a greater effectiveness than SS, but PTNS + SS was more effective than SS and PTNS. Furthermore, PTNS + SS showed a greater duration of effectiveness than PTNS and SS.

**Conclusions:**

Combination of PTNS with SS showed more effectiveness and more durability than PTNS and SS alone.

## INTRODUCTION

Overactive bladder syndrome (OAB) is a chronic disease characterized by urinary urgency with or without urge incontinence, frequency and nocturia (1) with huge economic burden and detrimental effects on the quality of life of patients (2). The prevalence of OAB in women increases with age and is present in approximately 30% of women over the age of 65 years (3) and in the general population it is estimated at 11.8% (4). Antimuscarinic agents represent the therapy of choice for the management of OAB, when the conservative treatment is ineffective, but although a long-lasting therapy is required to obtain better control of symptoms, a high percentage of patients discontinue the treatment after a few months (5, 6). After failure of a single drug, most practitioners try a different pharmacological treatment and if this second option also fails, more invasive options are considered (7). In recent years several studies have shown that a combination of two different anticholinergic or one anticholinergic combined with the β3-adrenoceptor agonist Mirabegron may improve efficacy in the treatment of OAB symptoms while also reducing the side effects (8). Furthermore, although the available data does not allow definitive evaluation (9), promising results were observed with an anticholinergic combined with bladder training, electric stimulation or percutaneous tibial nerve stimulation (10-12). The aim of this study was to assess the efficacy and the long term durability of Solifenacin (SS) and percutaneous tibial nerve stimulation (PTNS) administered alone or in combination in the treatment of women with OAB syndrome.

## MATERIALS AND METHODS

From May 2015 to December 2015, 105 consecutive women (mean age 61 years, range 41-73) with symptoms of overactive bladder (urgency, urinary frequency, with or without urge incontinence) were enrolled in this prospective, randomized, controlled study. Women were divided randomly into three groups of 35 patients each using online randomization (Graph Pad Quick Calcs software: http://www.graphad.com/quickcalcs/randomize1) by an independent biostatistician who was unaware of treatments performed by patients and was not involved in the study. In group-A, women received Solifenacin Succinate (SS) 5mg once a day for 12 weeks. In Group-B, women underwent PTNS once a week for 30 min each for a total of 12 weeks. In Group-C, patients underwent PTNS once a week for 30 min each for a total of 8 weeks and SS 5mg on alternate days also for 8 weeks (short-low dose therapy). The treatment was performed with a 34-gauge needle electrode inserted 6-8 centimeters cephalad to the medial malleolus and slightly posterior to the tibia. The electrode was connected to the Urgent PC stimulator and a current level of 0.5 to 9mA at 20Hz was selected based on patient sensory and motor response. Patients with urinary tract infection, neurological disease, bladder lithiasis, genital prolapse higher than stage II on POP-Q system, uncontrolled narrow angle glaucoma, pelvic tumours, post void residual urine ≥100mL, previously treated with radiation therapy, antimuscarinic agents, antidepressants and antianxiety agents, were excluded.

### Endpoints

Improvements in OAB symptoms, including day time frequency, night time frequency, urgency and urge incontinence were considered the primary efficacy end point. The impact of OAB symptoms on patient's quality of life (QoL) and the patient impression of improvement were considered the secondary end point. The primary efficacy end point was evaluated with Overactive Bladder Symptom Score (OABSS) questionnaire. It consists of 4 items related to OAB syndrome symptoms. The score of the first item (day time frequency) ranges from 0 to 2; the score of the second item (night time frequency) ranges from 0 to 3; the scores of the third and fourth items (urgency and urge incontinence respectively) range from 0 to 5. A greater score represents worsening of symptoms. The secondary end point was assessed with the Overactive Bladder questionnaire Short Form (OAB-q SF). The questionnaire consists of 6 items related to symptoms with 6 possible options ranging from “not at all” (score 1) to “a very great deal” (score 6), and a health-related quality of life scale with 13 items, with 6 response options ranging from “none of the time” (score 1) to “all of the time” (score 6). Improvement was evaluated with the Patient Global Impression of Improvement questionnaire (PGI-I). The PGI-I is a validated generic tool for assessment of the overall improvement or deterioration that patients experience following the treatment. It is a 7-point scale from “very much improved” (score 1), to “very much worse” (score 7). OABSS and PGI-I were performed before, at the end of each treatment and thereafter every month with a maximum follow-up of 10 months. OAB-q SF was performed before and after treatment. The study was conducted in accordance with the ethical principles of the Declaration of Helsinki and the protocol was approved by the local ethics committee. All patients signed informed consent before starting treatment. The results were assessed by a researcher blinded to treatment performed by patients.

### Statistical analysis

Statistical analysis was performed using the MedCalc software (version 12.7.7). Data in each group was assessed by D'Agostino-Pearson test. After having checked the normal distribution, data was processed using parametric tests: paired sample t test or independent sample t test. If normality was rejected, data was processed using non-parametric tests: Wilcoxon test for paired sample and Mann-Whitney for independent sample. Data was expressed as means±SD. A p value <0.05 was considered significant.

## RESULTS

Of the 35 women initially enrolled in group A, 8 patients suspended therapy because of side effects (dry mouth, constipation); therefore, 27 patients (mean age 62 years, range 41-70) were evaluable for the study. In Group-B, only one woman suspended therapy before the end of treatment without a specific reason; therefore. 34 patients (mean age 60 years, range 45-73) were evaluable for the study. In Group-C, two women suspended therapy: one because of side effects and one without a specific reason; therefore, 33 patients (mean age 63 years, range 43-72) were evaluable. Improvements with statistically significant differences were found in all the items assessed with OABSS questionnaire in the three groups of patients: day time frequency, night time frequency, urgency and urge incontinence (Table-1). No statistical difference was found between SS versus PTNS in daily and night time micturition, whereas a significant difference was found between combination therapy versus SS (P value: 0.0167). No significant difference was found between SS versus PTNS in urgency and urge incontinence, whereas significant differences were found in those items between the group of patients who underwent combination therapy and women treated with SS and women treated with PTNS (P values: 0.0005; 0.0225 and 0.0003; 0.0015 respectively) (Table-1). Significant improvements in quality of life of patients (OAB-q SF, 6 and 13 items) were found after treatment in all groups evaluated. However, no significant difference was found between SS versus PTNS, but combination therapy showed statistically significant differences both versus SS and versus PTNS (P values: 0.0168; 0.0287 and 0.0049; 0.0561 respectively) (Table-2). Improvements in PGI-I assessed at the end of treatment were found in all groups of patients. In any case, no significance was found between SS versus PTNS, whereas combination therapy showed significant improvements both versus SS and versus PTNS (P values: 0.0017; 0.0468 respectively). Evaluation of long term durability showed persistence of improvements of SS, PTNS and combination therapy assessed with OABSS questionnaire, respectively for 0.9, 2.5 and 5.9 months; the persistence of the improvements assessed with PGI-I questionnaire was respectively 0.7, 2.1, 5.6 months (Table-3). Statistical analysis showed a significant difference respectively in PTNS versus SS, in combination therapy versus SS and versus PTNS (p values: 0.0039; <0.0001; 0.0009 respectively) (Table-3).

**Table 1:** OAB symptoms assessed with OABSS questionnaire in patients treated with Solifenacin (SS), Percutaneous Tibial Nerve Stimulation (PTNS) and Solifenacin + Percutaneous Tibial Nerve Stimulation (SS + PTNS) at the end of treatment.

Patients N°	SS			PTNS			SS + PTNS		
	27			34			33		
Age	62(41-70)			60 (45-73)			63 (43-72)		
	before	after	p value	before	after	p value	before	after	p value
OABSS	1.19±0.39	0.71±0.45	0.0020*	1.24±0.42	0.47±0.50	<0.0001**	1.22±0.42	0.33±0.47	<0.0001*
Day-time	SS after		vs	PTNS after		0.1334***			
frequency	SS after		vs	SS + PTNS after		0.0167***			
	PTNS after		vs	SS + PTNS after		0.4224***			
Night-time frequency (score 0-3)	2.62±0.79	1.76±1.31	0.0078*	2.71±0.67	1.41±1.19	0.0010*	2.89±0.31	0.89±0.87	<0.0001*
	SS after		vs	PTNS after		0.4110***			
	SS after		vs	SS + PTNS after		0.0243***			
	PTNS after		vs	SS + PTNS after		0.1585***			
Urgency (score 0-5)	4.29±0.63	3.43±1.09	0.0002*	4.35±0.59	3.00±1.14	<0.0001**	4.44±0.68	2.11±0.99	<0.0001**
	SS after		vs	PTNS after		0.2586***			
	SS after		vs	SS + PTNS after		0.0005***			
	PTNS after		vs	SS + PTNS after		0.0225***			
Urge incontinence (score 0-5)	3.71±1.12	2.67±1.49	0.0005*	4.00±0.69	2.24±1.35	0.0001*	3.44±1.5	0.56±1.26	<0.0001*
	SS after		vs	PTNS after		0.3742***			
	SS after		vs	SS + PTNS after		0.0003****			
	PTNS after		vs	SS + PTNS after		0.0015****			

* Wilcoxon test (paired samples);

** Paired samples t-test;

*** Independent samples t-test;

**** Mann-Whitney test (independent samples)

**Table 2:** Improvements assessed with Quality of Life questionnaire (6 and 13 items) (OAB-q SF 6 and 13) and Patient Global Impression of Improvement questionnaire (PGI-I) at the end of treatment.

	SS			PTNS			SS + PTNS		
	before	after	p value	before	after	p value	before	after	p value
OAB-q SF 6	3.85±0.59	3.08±0.95	0.0001*	4.08±0.32	2.98±0.79	<0.0001*	4.21±0.24	2.32±0.87	<0.0001*
	SS after		vs	PTNS after		0.7525**			
	SS after		vs	SS + PTNS after		0.0168**			
	PTNS after		vs	SS + PTNS after		0.0287**			
OAB-q SF 13	3.92±0.62	3.16±0.95	0.0001*	4.22±0.32	2.96±0.97	<0.0001*	4.04±0.24	2.27±0.84	<0.0001*
	SS after		vs	PTNS after		0.5318**			
	SS after		vs	SS + PTNS after		0.0049**			
	PTNS after		vs	SS + PTNS after		0.0361**			
PGI-I		2.81±0.96			2.41±0.84			1.83±0.76	
	SS		vs	PTNS		0.1997**			
	SS		vs	SS + PTNS		0.0017**			
	PTNS		vs	SS + PTNS		0.0468**			

* Paired samples t-test

** Independent samples t-test

**Table 3:** Persistence of improvements in months, assessed with Overactive Bladder Symptoms Score (OAB-SS) and Patient Global Impression of Improvement (PGI-I) questionnaire (follow-up of 10 months).

	SS		PTNS		SS + PTNS
OAB-SS	0.93±1.44		2.50±2.38		5.88±2.20
	Solifenacin	vs	PTNS	p	0.0039*
	Solifenacin	vs	Solifenacin + PTNS	p	<0.0001*
	PTNS	vs	Solifenacin + PTNS	p	0.0009*
PGI-I	0.71±1.13		2.10±1.92		5.63±2.00
	Solifenacin	vs	PTNS	p	0.0017*
	Solifenacin	vs	Solifenacin + PTNS	p	<0.0001*
	PTNS	vs	Solifenacin + PTNS	p	0.0002*

* Mann-Whitney test (independent samples)

## DISCUSSION

The study showed a significant effectiveness with improvement in all parameters evaluated with OABSS questionnaire at the end of treatment, with all the treatments performed. Combination therapy was always statistically more effective than SS and it showed a significant improvement in urgency and urge incontinence, also when compared to PTNS. Improvement in women’ quality of life was observed in all groups assessed. However, combination therapy was statistically more effective than SS and PTNS. PGI-I evaluated at the end of the treatments showed an increase in perception of improvement in all groups of women. However, combination therapy was statistically more effective compared to SS and PTNS. We considered this PTNS + SS combination therapy, a short low dose treatment. The therapy was short due to the number of PTNS treatments performed (8 treatments instead of the usual 12) and the therapy was low dose due to the administration of SS on alternate days for 8 weeks. In addition to its effectiveness, this therapeutic scheme was developed to obtain a greater adherence especially in regard to a reduction in side effects and cost.

Studies performed on combination therapy in patients with OAB showed its effectiveness without demonstrating an increase in side effects: Bolduc et al., Kosilov et al. (7, 13), assessed combination of two anticholinergic drugs; Abrams et al. (8) assessed the combination of an anticholinergic with the β3-adrenoceptor agonist Mirabegron. Mattiasson et al. (10) combined tolterodine with simplified bladder training with an improvement on voiding frequency and voided volume; Curran et al. (14) achieved better results by combining anticholinergic with behavioural modifications; Souto et al., Kikilyel et al. (11, 12), assessed combination of electrical stimulations of the posterior tibial nerve versus respectively, oxybutinin and tolterodine. Amend et al. (15) and Abrams et al. (8) explained the success respectively of the combination of two anticholinergic drugs and of an anticholinergic with the β3-adrenoceptor agonist Mirabegron, by different receptor selectivity and by receptor interaction on different bladder wall regions. Improvements observed with the combination of PTNS with SS in this study can be explained by the blocking of muscarinic receptors that are responsible for vesical contractions caused by Solifenacine and by the effect of neuromodulation. The mechanism of bladder neuromodulation, although not completely clear, is different from that of drugs: its effect is possibly mediated through a combination of increasing cerebral endorphins, stimulation of somatic sacral and lumbar afferent fibers, and activation of efferent fibers to the striated urethral sphincter. The result of this possible mechanism is the inhibition of detrusor activity (16). The synergic effect of the combination therapy makes it possible to decrease the dose of each drug delivering an improved tolerability profile compared with monotherapy without compromising efficacy (13-8) with a greater adherence by the patient to therapy allowing its use even in elderly patients (8). In this study, we assessed with particular attention the duration of effectiveness of treatments performed. SS showed a short therapeutic efficacy with a mean of improvement after discontinuation of therapy of 0.9 months. A meta-analysis conducted by Rai et al. (9) of 23 trials of anticholinergic drugs versus non-drug active therapies for non-neurogenic overactive bladder syndrome, about the permanence of the therapeutic effect of anticholinergic drugs, emphasized how “it is unlikely that the effects of anticholinergics persist after stopping treatment”. PTNS administered in monotherapy or in combination with SS showed a longer duration of effectiveness with a mean of improvement after discontinuation respectively of 2.5 and 5.8 months. A longer duration of effectiveness of PTNS was described by MacDiarmid et al. (17). Therefore, combination therapy showed the longest therapeutic efficacy. Souto et al. assessed the effectiveness of TENS and oxybutynin alone or in combination therapy, underlining that TENS alone or in association presented longer lasting results than oxybutynin monotherapy. The choice of therapy should be based on several factors including co-morbidities, mental status, age, education and motivation (11). However, in clinical practice, antimuscarinic agents are considered the first-line pharmacotherapy for OAB, although a high percentage of patients discontinue the treatment after a few months especially for poor efficacy, side effects and costs (5, 6). Therefore, the search for a therapeutic strategy that improves adherence of patients to treatment depends on its efficacy and the reduction of side effects and cost.

## CONCLUSIONS

The combination of SS and PTNS in the treatment of OAB symptoms was an effective and well-tolerated therapy with greater improvements and longer lasting effects than single treatments. It allowed the use of lower amounts of drug with a smaller number of PTNS treatments. The low sample size of patients and the follow-up of ten months represent biases that do not allow definitive conclusions to be drawn. Further studies on a greater number of patients would be desirable to confirm this data.
